# Isolation and Characterization of Vancomycin-Resistant *Enterococcus faecium* from Cattle: Antimicrobial Resistance, Virulence Genes, and Pathogenicity

**DOI:** 10.3390/vetsci12090880

**Published:** 2025-09-12

**Authors:** Mengyuan Cao, Fang Cao, Chenyu Wang, Xueqi Yan, Feng Dong, Shilei Zhang, Shaymaa Abousaad, Lin Yang, Ayman M. Abouzeid, Yongjie Wang, Yayin Qi

**Affiliations:** 1 College of Animal Science and Technology, Shihezi University, Shihezi 832003, China; cmy1110@163.com (M.C.); 15666429308@163.com (F.C.); 18899532722@163.com (C.W.); 18899531882@163.com (X.Y.); laoshouyi1984@163.com (F.D.); sz020@shzu.edu.cn (S.Z.); 2 Department of Animal Sciences, College of Agriculture and Environmental Sciences, North Carolina Agricultural and Technical University, Greensboro, NC 27411, USA; smabousaad@ncat.edu (S.A.); lyang124@aggies.ncat.edu (L.Y.); 3 Department of Biology, College of Science and Technology, North Carolina Agricultural and Technical State University, Greensboro, NC 27411, USA; 4 Department of Agribusiness, Applied Economics and Agriscience Education, College of Agriculture and Environmental Science, North Carolina Agricultural and Technical State University, Greensboro, NC 27411, USA; amabouzeid@ncat.edu

**Keywords:** *Enterococcus faecium* (*E. faecium*), vancomycin, biochemical identification, antimicrobial resistance, virulence gene profile, pathogenicity

## Abstract

This study aimed to investigate the antimicrobial resistance, virulence gene profile, and pathogenicity of *Enterococcus faecium* isolated from a large-scale cattle farm in Xinjiang, China, to provide a scientific basis for the prevention and control of *E. faecium* infections in the region. Nineteen rectal swabs were aseptically collected from diarrheic calves for bacterial isolation. Isolates were identified through morphological observation, biochemical characterization, and PCR amplification. Homology analysis was conducted using 16S rRNA gene sequencing. Antimicrobial susceptibility was evaluated by the disk diffusion method, and key virulence genes were detected using PCR. Pathogenicity was assessed through intraperitoneal inoculation of mice, followed by histopathological examination. Three isolates were identified as *E. faecium*, consistent with morphological and molecular results. Biochemical tests indicated that the strains could metabolize sucrose, maltose, lactose, melibiose, and raffinose, but not sorbitol. Phylogenetic analysis revealed that isolates SCQ3 and SCQ4 shared 99.3% homology with *E. faecium* strain MF678878.1, while SCQ11 showed 91% similarity to strain JP2. Antimicrobial susceptibility testing indicated that SCQ11 was resistant to vancomycin. PCR analysis identified the presence of multiple virulence genes, including *psaA*, *hyp*, *asal*, *sprE*, *nuc*, *cbh*, *srtA*, *hyl*, *scm*, and *agg*. In vivo pathogenicity testing demonstrated that the vancomycin-resistant strain exhibited strong virulence in mice, with gross lesions observed in the liver, spleen, and intestines. Histopathological examination confirmed varying degrees of tissue damage, particularly in the liver and spleen. All three *E. faecium* isolates exhibited multidrug resistance, with one strain showing vancomycin resistance and harboring a high number of virulent genes. This strain demonstrated significant pathogenicity in vivo. These findings highlight the potential public health threat posed by multidrug-resistant *E. faecium* in livestock and provide essential data for regional prevention and control strategies.

## Simple Summary

Antibiotic resistance is a growing problem that threatens both human and animal health. In this study, we investigated bacteria called *Enterococcus faecium*, which are normally found in the intestines of animals but can sometimes cause serious disease. We collected samples from calves with diarrhea on a cattle farm in Xinjiang, China, and identified three strains of *E. faecium*. One of these strains was resistant to vancomycin, a very important antibiotic often used as a last resort in hospitals. This resistant strain also carried many genes that help bacteria cause disease, such as those that enable them to stick to host tissues, avoid the immune system, and damage organs. When tested in mice, this strain caused illness and visible damage to the liver and spleen. Our findings show that drug-resistant *E. faecium* in livestock can pose risks not only to animal health but also to public health, since these strains may spread to humans or the environment. This study highlights the importance of monitoring resistant bacteria in farm animals and developing strategies to reduce their spread, which will help protect both animal farming and society at large.

## 1. Introduction

*Enterococcus faecium* is a Gram-positive coccus belonging to the genus *Enterococcus* that is widely distributed in environmental sources such as soil and water and forms part of the normal gut microbiota of humans and animals [1]. Although many strains are harmless and used as probiotics in both human and veterinary contexts [2,3,4,5], *E. faecium* has emerged as a significant opportunistic pathogen capable of causing a range of infections in both humans and animals [6].

In livestock systems, particularly in cattle, infection rates of *E. faecium* are also rising [7]. A major contributing factor is the widespread and sometimes inappropriate use of antibiotics in animal agriculture, which creates selective pressure that favors the survival and spread of resistant strains [8]. This practice has accelerated the emergence of multidrug-resistant (MDR) *E. faecium*, raising serious concerns about their potential to be transmitted to humans through direct contact, the food chain, or the environment. While some animal-derived E. faecium strains may lack virulence determinants, others carry genetic elements that enhance their ability to cause disease and complicate treatment outcomes [9]. These dynamics underscore the need for ongoing molecular surveillance and robust antibiotic stewardship programs to curb further resistance development [3,10,11].

In recent years, *E. faecium* has transitioned from a benign commensal organism into a high-risk, MDR hospital-associated pathogen. This transformation is largely attributed to its remarkable genetic plasticity, which allows rapid acquisition of antimicrobial resistance (AMR) genes through mutation and horizontal gene transfer, often via plasmids [12,13,14,15]. Some strains have even been shown to release membrane vesicles containing resistance proteins, further facilitating the spread of resistance [16]. Clinically, *E. faecium* exhibits both intrinsic resistance to many first-line antibiotics (e.g., cephalosporins, aminoglycosides) and acquired resistance to critical agents like ampicillin [13,14,17,18]. While resistance to linezolid and daptomycin remains less common, it is increasingly reported [19,20]. These resistance genes are frequently plasmid-borne, facilitating their spread across clonal lineages and host species [12,14]. Hospital-adapted *E. faecium* lineages, such as A1 and ST796, carry numerous resistance and virulence genes and exhibit strong environmental resilience [12,14,21]. Their tolerance to desiccation and disinfectants enables persistence in clinical settings [13,15,22], contributing to nosocomial infections such as bloodstream, urinary tract, and wound infections, especially in immunocompromised patients [15,17,23]. Importantly, *E. faecium* is also present in livestock, including cattle [5,24,25,26]. In these animals, resistance to critically important antibiotics such as tetracyclines, macrolides, and other antimicrobial classes is frequently observed [25,26,27,28,29,30,31]. Moreover, the occasional overlap in sequence types between bovine and human isolates raises concerns about potential zoonotic transmission or shared reservoirs [30,32,33]. These findings highlight the importance of continued genomic surveillance and careful probiotic use in livestock to minimize public health risks.

Resistance trends in *E. faecium* vary across regions and over time, shaped by local antibiotic usage and infection control measures [5,11]. As such, molecular surveillance and antibiotic stewardship are critical to limiting the spread of resistant clones [3,10,11].

To better understand the resistance mechanisms and virulence potential of *E. faecium* in livestock, this study serves as a model investigation. Rectal swabs were collected from diarrheic calves on a large-scale cattle farm in Xinjiang, China. *E. faecium* strains were isolated, purified, and identified. Antimicrobial susceptibility testing and virulence gene detection were performed, and pathogenicity was evaluated via intraperitoneal inoculation in mice. The results provide a scientific foundation for the prevention and control of *E. faecium* infections in cattle and contribute to the broader discussion on their impact on public and animal health.

## 2. Materials and Methods

### 2.1. Sample Collection and Isolation and Identification of E. faecium

A total of 19 rectal swabs were aseptically collected from diarrheic calves on a cattle farm in Xinjiang Province, China. Swabs were placed in transport media and stored at 4 °C before processing within 24 h. According to the previous procedure [34], each swab was inoculated into brain heart infusion (BHI) broth under sterile conditions and incubated overnight at 37 °C. A small volume of the overnight culture was used for Gram staining and microscopic examination. Cultures were then streaked onto Pfizer enterococcus selective agar plates (Pfizer Inc., Shanghai, China) and incubated at 37 °C for 18–24 h. Colonies appeared as brown/black with brown halos, and the colony morphology was observed and recorded. Typical single colonies from the selective agar plates were picked and sub-cultured in BHI broth, followed by staining and microscopic examination to observe bacterial morphology. The cultures were then streaked onto blood agar plates and incubated at 37 °C for 18–24 h to further assess colony morphology.

Physiological and biochemical identification was performed using bacterial micro biochemical identification tubes (Hope Bio-Technology Co., Ltd., Qingdao, Shandong, China) following *Bergey’s Manual of Systematic Bacteriology protocols* for *Enterococcus* species. The strains tested positive for arginine dihydrolase, fermented multiple carbohydrates, hydrolyzed esculin, and showed expected TMZ and DPP positive, MAG and PMG negative reactions, consistent with *E. faecium*.

Genomic DNA was extracted using the boiling method. PCR amplification of the *E. faecium*-specific *ddl* gene was performed using the following primer pair: *ddl*-F: ACGTTGGATGTCGGCATTACAAAGG and *ddl*-R: ACGTTGGATGAAGTCGTCCGAACAT, with an expected product size of 176 bp. The PCR reaction mixture consisted of 2 µL DNA template, 1 µL of each forward and reverse primer, 10 µL of 2× Taq PCR Mix, and nuclease-free water to total 20 µL volume. The thermal cycling conditions were as follows: initial denaturation at 95 °C for 30 s; 30 cycles of denaturation at 95 °C for 30 s, annealing at 55 °C for 30 s, and extension at 72 °C for 30 s; followed by a final extension at 72 °C for 10 min and a hold at 4 °C.

For species confirmation, 16S rRNA gene amplification and sequencing were performed using universal primers to yield a ~1500 bp product [35]. PCR products were gel-purified and sequenced using Sanger sequencing. The obtained sequences were subjected to BLAST+ 2.16.0 analysis against the NCBI nucleotide database to assess sequence similarity. A phylogenetic tree was constructed based on the aligned 16S rRNA sequences to evaluate genetic relationships among isolates using MEGA 10 software and the neighbor-joining method with 1000 bootstrap replicates.

Three strains were selected from the isolated and purified *Enterococcus faecalis* strains for subsequent pathogenicity testing. The selection criteria were as follows: From 19 rectal swab samples, these three strains were isolated from three calves with typical clinical signs of diarrhea (defined as ≥3 watery fecal episodes per day) and no other co-infections. Additionally, the colony detection density of these strains on selective agar plates was significantly higher than that of other isolates at the time of isolation.

### 2.2. Antimicrobial Susceptibility Testing

Antimicrobial susceptibility was tested for SCQ3, SCQ4, and SCQ11 using the Kirby–Bauer disk diffusion method, following the guidelines of the Clinical and Laboratory Standards Institute [36]. A total of 14 antibiotics were tested: azithromycin (15 µg), streptomycin (10 µg), roxithromycin (30 µg), vancomycin (30 µg), kanamycin (30 µg), gentamicin (10 µg), tetracycline (30 µg), enrofloxacin (5 µg), ciprofloxacin (5 µg), nitrofurantoin (300 µg), cefotaxime sodium (30 µg), oxacillin (1 µg), chloramphenicol (30 µg), and sulfadiazine (300 µg). The antimicrobial resistance profiles of the isolates were determined based on the diameters of the inhibition zones, as interpreted according to CLSI standards [36]. Quality control strain *E. faecalis* ATCC 29212 was used.

### 2.3. Virulence Gene Detection

PCR was used to detect the presence of virulence genes including *psaA*, *hyp*, *asal*, *sprE*, *nuc*, *cbh*, *srtA*, *hyl*, *scm*, *ace*, and *agg*. The primer sequences and expected amplicon sizes are listed in Table 1. The PCR conditions were as follows: initial denaturation at 95 °C for 5 min; followed by 30 cycles of denaturation at 95 °C for 30 s, annealing at 55 °C for 35 s, and extension at 72 °C for 45 s; with a final extension at 72 °C for 10 min. Amplified products were separated by electrophoresis on a 1% agarose gel at 120 V for 30 min and visualized using a gel imaging system were visualized under UV after staining with ethidium bromide. Positive controls for each virulence gene and nuclease-free water as negative control were included. Bands were compared to a 100 bp DNA ladder.

### 2.4. Animal Pathogenicity Assay

The animal study protocol was approved by the Biology Ethics Committee of Shihezi University in March 2024 (A2024-136).

Ten C57BL/6 mice (6–8 weeks old) were randomly divided into two groups (n = 5 per group). Mice in the experimental group were intraperitoneally inoculated with 0.2 mL of the bacterial suspension, while those in the control group received an equal volume of sterile PBS. Clinical symptoms and survival were monitored for 72 h. At the end of the experiment or upon death, liver, spleen, and intestines were aseptically harvested. Bacteria were re-isolated, and *E. faecium* was confirmed via *ddl* PCR (176 bp product) using 2× Taq PCR Master Mix (Tiangen Biotech Co., Ltd., Beijing, China). Tissues were fixed in 10% formalin, embedded in paraffin, sectioned at 5 µm, and stained with Hematoxylin and Eosin (H&E). Lesions were assessed under light microscopy.

### 2.5. Statistical Analysis

Descriptive analysis was used to interpret antimicrobial susceptibility profiles based on inhibition zone diameters for individual isolates. Sensitivity classifications were assigned according to Clinical and Laboratory Standards Institute criteria [36]. For virulence gene detection and biochemical test results, presence or absence was recorded descriptively. Pathogenic outcomes, including clinical observations and histopathological findings, were qualitatively compared between groups. Statistical testing was not applied due to the categorical nature of the data.

## 3. Results

### 3.1. Isolation and Identification Pathogenic Strains of E. faecium

**Figure 1:**
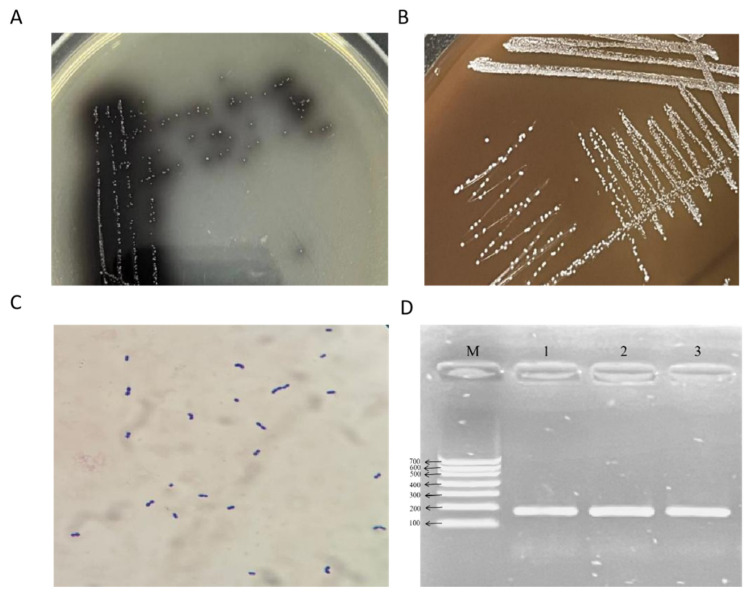

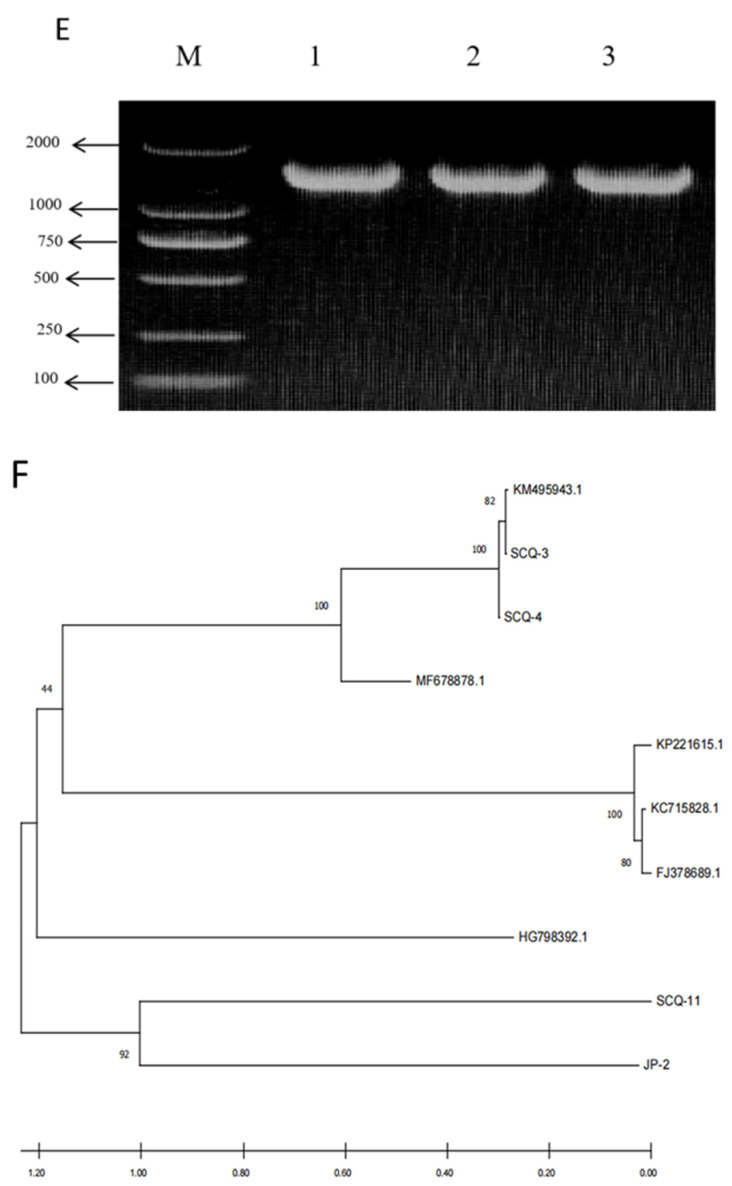
Identification and molecular characterization of *E. faecium* isolates. (**A**) Colony morphology on Enterococcus-selective agar showing brown-black colonies with brown halos. (**B**) Colony morphology on blood agar with small, round, slightly raised, grayish-white, translucent colonies. (**C**) Gram staining showing Gram-positive cocci, occurring singly or in pairs. (**D**) PCR amplification of the *ddl* gene showing a 176 bp product specific to *E. faecium*; Lanes 1–3 in correspond to isolates SCQ3, SCQ4, and SCQ11, respectively. (**E**) 16S rRNA gene amplification producing an approximately 1500 bp band; Lanes 1–3 in correspond to isolates to SCQ3, SCQ4, and SCQ11, respectively. (**F**) Neighbor-joining phylogenetic tree constructed using MEGA X from aligned 16S rRNA sequences, illustrating genetic relationships between isolates and reference *E. faecium* strains from NCBI. Bootstrap values (n = 1000 replicates) shown at branches; scale bar indicates evolutionary distance.

A total of three pathogenic *E. faecium* strains were isolated from 19 collected samples. After overnight incubation in BHI broth, smears of the cultures were subjected to Gram staining, which revealed numerous Gram-positive cocci under microscopic examination. When streaked onto Enterococcus-selective agar, the isolates produced brown-black colonies surrounded by brown halos (Figure 1A). On blood agar plates, the colonies appeared as small, round, slightly raised, grayish-white, translucent colonies with smooth and moist surfaces (Figure 1B). Gram staining again confirmed that all isolates were Gram-positive cocci, occurring singly or in pairs (Figure 1C).

Colony morphology and staining results were consistent with typical *E. faecium* characteristics. PCR amplification targeting the *E. faecium*-specific *ddl* gene produced a clear band of approximately 176 bp (Figure 1D). Lanes 1–3 in Figure 1D correspond to isolates SCQ3, SCQ4, and SCQ11, respectively. Amplification using universal 16S rRNA primers yielded a product of approximately 1500 bp (Figure 1E). Lanes 1–3 in Figure 1E also correspond to SCQ3, SCQ4, and SCQ11.

The amplified 16S rRNA fragments were gel-purified and sequenced. Sequence analysis revealed that isolates SCQ3 and SCQ4 shared 100% sequence identity and were therefore considered the same strain. SCQ3/SCQ4 showed 99.3% sequence similarity to strain MF678878.1 and clustered in the same phylogenetic branch. Isolate SCQ11 exhibited 91% similarity with strain JP2 and grouped within the same branch (Figure 1F). The phylogenetic tree confirmed the genetic relatedness of the isolates to known *E. faecium* strains, validating their identification.

The biochemical identification results of the isolates are shown in Table 2. The strains tested positive for the arginine dihydrolase test and were able to ferment fructose, melibiose, xylose, trehalose, maltose, glucose, lactose, sucrose, and raffinose, but not sorbitol. They were also capable of hydrolyzing esculin. Positive reactions were observed in TMZ and DPP tests, while MAG and PMG tests were negative. These results are consistent with the biochemical characteristics of *Enterococcus faecium*. All three isolates exhibited a similar biochemical profile consistent with *E. faecium*, further supporting the molecular identification.

### 3.2. Antimicrobial Susceptibility Testing of Isolates

Antimicrobial susceptibility testing was conducted for isolates SCQ3, SCQ4, and SCQ11. The results showed that SCQ3 was highly sensitive to azithromycin, roxithromycin, vancomycin, nitrofurantoin, and chloramphenicol, and moderately sensitive to enrofloxacin and tetracycline. SCQ4 exhibited extreme sensitivity to vancomycin and nitrofurantoin, high sensitivity to azithromycin, roxithromycin, enrofloxacin, and chloramphenicol, and moderate sensitivity to tetracycline. In contrast, SCQ11 showed high sensitivity only to nitrofurantoin and moderate sensitivity to azithromycin but was not sensitive to vancomycin or most other antibiotics tested. These results indicate a varied AMR pattern among the three isolates, with SCQ11 exhibiting a broader resistance profile compared to SCQ3 and SCQ4. The detailed antimicrobial susceptibility profiles are presented in Table 3.

### 3.3. Virulence Gene Detection in Vancomycin-Resistant Isolate SCQ11

**Figure 2:**
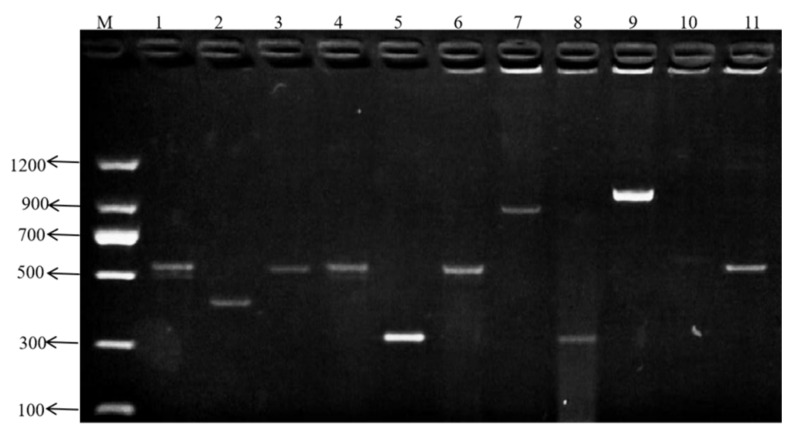
PCR amplification results for virulence gene detection in *E. faecium* isolates. M: DNA ladder (100 bp marker); Lanes 1–11: PCR amplicons for virulence genes. 1: *psaA* gene; 2: *hyp* gene; 3: *asal* gene; 4: *sprE*; 5: *nuc* gene; 6: *cbh* gene; 7: *srtA* gene; 8: *hyl* gene; 9: *scm* gene; 10: *ace* gene (not detected); 11: *agg* gen. Bands indicate the presence of the corresponding gene.

PCR was used to detect the presence of 11 major virulence genes in the vancomycin-resistant strain SCQ11. As shown in Figure 2, a total of 10 virulence genes were detected, including *psaA*, *hyp*, *asal*, *sprE*, *nuc*, *cbh*, *srtA*, *hyl*, *scm*, and *agg*. The *ace* gene was not detected. These findings demonstrate that SCQ11 harbors a broad array of virulence-associated genes in addition to extensive antimicrobial resistance, underscoring its clinical significance as a multidrug-resistant pathogen.

### 3.4. Pathogenicity Assessment of Vancomycin-Resistant Isolate SCQ11

**Figure 3:**
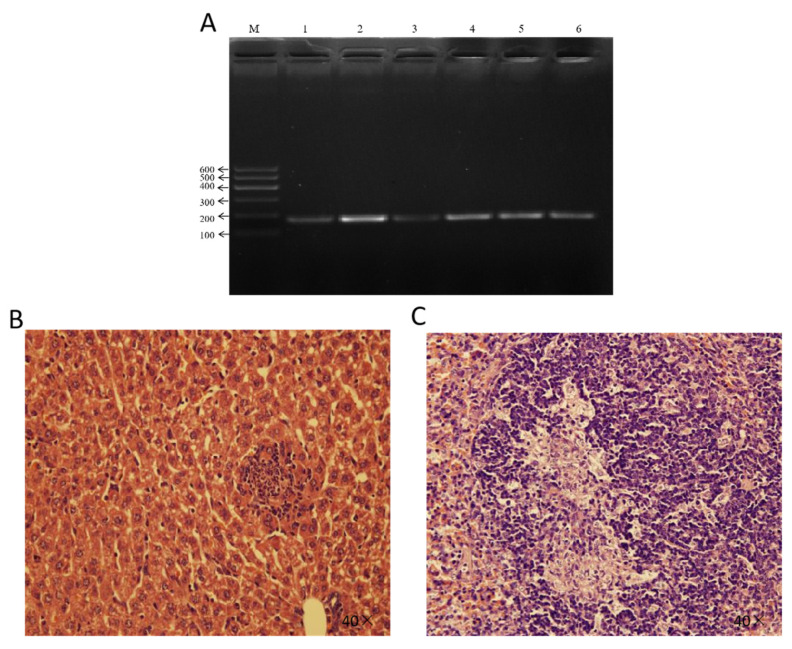
Pathogenicity assessment of *E. faecium* isolate SCQ11 in mice. (**A**) PCR electrophoresis of enterococci-specific primers targeting the *ddl* gene (176 bp) in tissue samples from infected mice. M: DNA ladder; Lanes 1–6: 1: liver 1; 2: intestine; 3: brain; 4: spleen 1; 5: liver 2; 6: spleen 2. (**B**) H&E-stained liver section from an infected mouse showing congestion and cellular disorganization (40×). (**C**) H&E-stained spleen section from an infected mouse showing disruption of normal architecture and hemorrhagic infiltration (40×).

Mouse infection experiments revealed that mice in the experimental group exhibited varying degrees of lethargy piloerection, and reduced activity. Postmortem examination revealed congestion and hemorrhaging in the liver and spleen, along with swelling in the liver, spleen, and intestinal tissues. Tissue samples from deceased mice were aseptically inoculated into liquid culture media, and bacterial isolation and identification confirmed the presence of *E. faecium*. PCR using *E. faecium*-specific primers yielded the expected 176 bp product (Figure 3A). Histopathological analysis of infected liver and spleen revealed hemorrhagic lesions in both the liver and spleen and distinct pathological alterations, including disorganized hepatic architecture with hemorrhagic foci and splenic tissue damage with lymphocyte depletion and hemorrhage (Figure 3B,C).

## 4. Discussion

*E. faecium* is a common member of the normal intestinal microbiota in both humans and animals [1,8], and certain strains have been used as probiotics due to their ability to inhibit pathogenic bacteria and enhance host immune function [2,3,4,5]. For example, it was reported that dietary supplementation with 1 × 10^7^ CFU/kg *E. faecium* for 28 days in mid-lactation Holstein dairy cows reduced lactate dehydrogenase levels, increased antitumor factor concentrations and red blood cell counts, and improved cellular immune responses [37]. However, under immunocompromised conditions, *E. faecium* may act as an opportunistic pathogen [6], translocating from the intestinal tract to other organs where it adheres, aggregates, and initiates infection [20,38,39], leading to serious diseases such as bacteremia and endocarditis [40]. Since the 1990s, enterococci have increasingly been recognized as significant opportunistic pathogens in both humans and animals, with infection cases rising annually [8]. The widespread misuse and overuse of antibiotics has further contributed to the emergence of drug-resistant strains.

In this study, three pathogenic *E. faecium* strains (SCQ3, SCQ4, and SCQ11) were isolated from rectal swabs of diarrheic calves on a cattle farm in Xinjiang Province, China. A limitation of this study is that strain isolation was conducted at a single location, which may restrict the representativeness of the samples. Therefore, future studies involving multiple geographic regions and larger sample sizes are needed to better understand the distribution, epidemiological significance, and broader impact of *E. faecium* in cattle populations. However, the core objective of this study was to validate the expression of specific virulence genes and the in vitro pathogenicity of the target pathogen, not to statistically determine the infection rate across different cattle populations. From these samples, the three selected strains were isolated from three calves with typical clinical signs of diarrhea (defined as ≥3 watery fecal episodes per day) and no other co-infections. Additionally, the colony detection density of these strains on selective agar plates was significantly higher than that of other isolates at the time of isolation. These strains were identified through Gram staining, biochemical profiling, and 16S rRNA PCR amplification. The use of combined morphological, biochemical, and molecular techniques minimized misidentification and confirmed the isolates as *E. faecium*. Phylogenetic analysis based on 16S rRNA sequences revealed that SCQ3 and SCQ4 shared 99.3% sequence identity with strain MF678878.1 and clustered in the same branch, while SCQ11 exhibited 91% similarity to strain JP2 and formed a separate cluster.

The pathogenicity of *E. faecium* is largely attributed to its resistance to multiple antibiotics and the presence of virulence genes [8]. Multidrug resistance makes treatment of *E. faecium* infections particularly challenging [41]. Therefore, determining the susceptibility profile is essential for guiding appropriate antimicrobial therapy [15]. In this study, antimicrobial susceptibility testing showed that SCQ3 was highly sensitive to azithromycin, roxithromycin, vancomycin, nitrofurantoin, and chloramphenicol; SCQ4 exhibited extreme sensitivity to vancomycin and nitrofurantoin, and high sensitivity to azithromycin, roxithromycin, enrofloxacin, and chloramphenicol. In contrast, SCQ11 was only highly sensitive to nitrofurantoin and moderately sensitive to azithromycin and was resistant to vancomycin and most other antibiotics. All three isolates demonstrated multidrug resistance, which may be associated with long-term or improper use of antibiotics on the farm, leading to compromised host immunity and increased pathogen virulence.

These findings are consistent with the well-documented trend of *E. faecium* acquiring multidrug resistance, particularly in agricultural settings where antibiotics may be used routinely or inappropriately [42,43]. The resistance profile of SCQ11, which includes vancomycin and multiple other antibiotic classes, raises concern about the potential for zoonotic transmission of resistant strains. Studies have shown that such resistance is often mediated by mobile genetic elements like plasmids and transposons, facilitating horizontal gene transfer across strains and host species [12,44]. The susceptibility of SCQ3 and SCQ4 to key antibiotics may reflect limited prior exposure or recent introduction into the farm environment, while SCQ11’s broader resistance could suggest a longer adaptation period or acquisition of resistance determinants through selective pressure. These patterns emphasize the importance of antimicrobial stewardship in livestock to prevent the emergence and dissemination of resistant *E. faecium* strains with enhanced pathogenic potential. The emergence of vancomycin-resistant *Enterococcus* strains (VRE) in livestock poses a significant public health threat, as these findings underscore a critical **One Health** concern where the health of animals and humans are inextricably linked. The potential for these resistant strains to be transmitted from livestock to humans—either directly through farm workers or indirectly via the food chain and environmental contamination—is well-documented. This transmission pathway compromises the efficacy of vancomycin, a last-resort antibiotic for treating severe human infections, particularly those caused by multidrug-resistant Gram-positive bacteria. Addressing this challenge requires an integrated, multi-sectoral approach that includes veterinary medicine, human medicine, and environmental science to implement robust antimicrobial stewardship programs [45,46,47,48].

In addition to antibiotic resistance, the pathogenicity of *E. faecium* is also closely linked to its acquisition of virulence genes [49]. These genes play a key role in the infectious process. Known virulence factors in *Enterococcus* species include the enterococcal surface protein gene (*esp*), which facilitates adhesion and biofilm formation; hyaluronidase gene (*hyl*), which degrades host connective tissue and aids dissemination; collagen-binding adhesin gene (*ace*), which promotes binding to extracellular matrix proteins; gelatinase gene (*gelE*), which hydrolyzes host proteins; cytolysin gene (*cyl*), which contributes to hemolysis and tissue damage [50]; and aggregation substance gene (*asa1*), and collagen adhesion protein gene (*agg*), which enhance intercellular adherence and plasmid transfer, promoting pathogenicity and resistance spread [51]. In this study, 10 virulence genes, *psaA*, *hyp*, *asal*, *sprE*, *nuc*, *cbh*, *srtA*, *hyl*, *scm*, and *agg*, were detected in the vancomycin-resistant isolate SCQ11; only the *ace* gene was not detected. Pathogenicity testing in mice revealed that SCQ11 had strong virulence, which may be due to the high number of virulence genes and potential synergistic effects among them. The detection of 10 virulent genes in the SCQ11 strain highlights its strong pathogenic potential. Genes such as *hyl* (involved in tissue degradation), *sprE* and *nuc* (involved in immune evasion), and *agg* (promoting adhesion and biofilm formation) may act synergistically to enhance colonization, invasion, and persistence in the host [49,51]. The absence of the ace gene, a collagen-binding protein commonly associated with endocarditis and urinary tract infections, may indicate a different pathogenic strategy in SCQ11, potentially favoring bloodstream or intestinal colonization routes. The presence of such a broad virulence repertoire, particularly in a vancomycin-resistant background, raises concern for zoonotic transmission and highlights the strain’s public health relevance. These genes are likely to contribute not only to disease severity but also to resistance to host immune clearance mechanisms. Moreover, the co-presence of multiple virulence genes and vancomycin resistance in SCQ11 suggests a concerning convergence of antimicrobial resistance and pathogenic potential. The ability of *E. faecium* to simultaneously harbor genes like *agg*, *hyl*, *srtA*, and *scm*, which are involved in adhesion, immune evasion, and tissue invasion, may enhance its fitness and adaptability in the host environment [17]. This combination is particularly alarming in the context of animal–human transmission, as it increases the likelihood that multidrug-resistant and virulent strains can colonize and infect immuno-compromised hosts. Several studies have suggested that virulence and resistance determinants may be co-selected through shared mobile genetic elements or induced under antibiotic pressure [44]. These findings underscore the need for integrated surveillance of both resistance and virulence traits in livestock-associated enterococcal populations to inform risk assessments and containment strategies.

## 5. Conclusions

This study successfully isolated and identified three *E. faecium* strains (SCQ3, SCQ4, and SCQ11) from diarrheic calves using bacteriological and molecular methods. Their antimicrobial resistance and pathogenicity were characterized, providing foundational data for future prevention and control strategies targeting *E. faecium*. With the continuous increase in virulence and resistance of *E. faecium*, its threat to animal husbandry is becoming more serious [52]. Further research is needed to elucidate the mechanisms underlying antibiotic resistance and virulent gene expression, which will be critical for developing effective strategies to mitigate *E. faecium*-associated infections in both animals and humans.

## Figures and Tables

**Table 1:** Primer sequences and amplicon sizes.

Gene(s)	Primer Sequence (5′-3′)	Size (bp)
*psaA*	CTATTTTGCAGCAAGTGATG	540
	CGCATAGTAACTATCACCATCTTG	
*hyp*	TAGCGAATAAAACAGTCACC	380
	AACTTGTGCTTGTCGAGAAA	
*asal*	CCAGCCAACTATGGCGGAATC	529
	CCTGTCGCAAGATCGACTGTA	
*sprE*	CGTTCCTGCCGAAAGTC	570
	GATTGGGGAACCAGATTGA	
*nuc*	GTGTAAAAGAAGTTACTGAAAATGTTACTC	332
	GCGTTTTTTGTAGTAATGTTCCATCTACG	
*Cbh*	CTCATAGGATCCATCACCAACATCAC	580
	TGGCTGGAATTCACTTTTCAGGCTAT	
*SrtA*	TTGGAATCTAGAAATAACACCTTCTTGCAAGATACCTTTC	876
	TTTTTTCTGCAGTGGGCGCATATTTTCCCTCCTTTTAATG	
*hyl*	ACAGAAGAGCTGCAGGAAATG	276
	GACTGACGTCCAAGTTTCCAA	
*scm*	GTTTACTAGTCCTAGTTGC	1015
	TCTGTACTGTCGCTTGTGTC	
*ace*	GGAATGACCGAGAACGATGGC	616
	GCTTGATGTTGGCCTCCTTCCG	
*Agg*	CACGTAATTCTTGCCCACCA	520
	CAAGCATTATTGGCAGCGTT	

**Table 2:** Biochemical identification results of *E. faecium* isolates.

Biochemical Test	Result	Biochemical Test	Result	Biochemical Test	Result
Sorbitol	−	PMG	−	Hydrogen sulfide	−
Fructose	+	Sucrose	+	Methyl Red (MR)	−
Phosphate Glucose Peptone Water	−	N-acetylglucosamine	+	Indole (Peptone Water)	−
Melibiose	+	DPP	+	Raffinose	+
Nitrate	−	Ornithine	+	Lysine	+
Urea	+	Mannitol	+	Gluconate	−
Xylose	+	Arginine	+	Adonitol	−
Trehalose	+	Esculin	+	Citrate	−
Maltose	+	MAG	−	TMZ	+
Mannitol	+	Amino acid utilization	+	Phenylalanine	−
Lactose	+	—	—	—	—

Note: “+” = positive reaction; “−” = negative reaction.

**Table 3:** Diameter of inhibition zones (mm) of three *E. faecium* isolates against tested antibiotics.

Antibiotic	SCQ3	AR	SCQ4	AR	SCQ11	AR
Azithromycin	15	S	17	S	10	I
Streptomycin	0	R	0	R	0	R
Roxithromycin	17	S	18	S	0	R
Vancomycin	17	S	20	S	0	R
Kanamycin	0	R	0	R	0	R
Gentamicin	0	R	0	R	0	R
Tetracycline	10	I	10	I	0	R
Enrofloxacin	10	I	17	S	0	R
Ciprofloxacin	0	R	0	R	0	R
Nitrofurantoin	18	S	20	S	16	S
Cefotaxime sodium	0	R	0	R	0	R
Oxacillin	0	R	0	R	0	R
Chloramphenicol	17	S	19	S	0	R
Sulfadiazine	0	R	0	R	0	R

Note: Interpretation of sensitivity based on inhibition zone diameter: ≥20 mm = extremely sensitive; 15–19 mm = highly sensitive; 10–14 mm = moderately sensitive; <10 mm = low sensitivity; 0 mm = resistant; AR: antimicrobial resistance; R: resistant; I: intermediate; S: susceptible.

## Data Availability

The original contributions presented in this study are included in the article. Further inquiries can be directed to the corresponding authors.

## Abbreviations

16S rRNA	16S Ribosomal Ribonucleic Acid
*ace*	Adhesin to Collagen of *Enterococcus*
*agg*	Aggregation Substance
AMR	Antimicrobial Resistance
*asa1*	Aggregation Substance Gene
*asal*	Aggregation Substance Associated Lipoprotein
ATCC	American Type Culture Collection
BHI	Brain Heart Infusion
*cbh*	Cell-Binding Homolog
*cyl*	Cytolysin Gene
CFU/kg	Colony-Forming Units per Kilogram
CLSI	Clinical and Laboratory Standards Institute
*esp*	Enterococcal Surface Protein Gene
*E. faecium*	*Enterococcus faecium*
*gelE*	Gelatinase Gene
H&E	Hematoxylin and Eosin
*hyl*	Hyaluronidase
*hyp*	Hypothetical Protein (unknown function)
MAG	Mannose Agar Gel Test
MDR	Multidrug-Resistant
*nuc*	Nuclease
NCBI	National Center for Biotechnology Information
PBS	Phosphate-Buffered Saline
PMG	Phenol-Mannitol Gel Test
PCR	Polymerase Chain Reaction
*psaA*	Pneumococcal Surface Adhesin A
SCQ3, SCQ4, SCQ11	Strain Codes for *E. faecium* Isolates
*scm*	Surface Protein (Collagen-binding Adhesin)
*sprE*	Serine Protease
*srtA*	Sortase A (anchors surface proteins to cell wall)
TMZ	Tryptone-Mannitol-Zinc Test
UV	Ultraviolet

## References

[1] Aziz F., Khan M.N., Ahmed S., Andrews S.C. (2019). Draft Genome Sequence of *Enterococcus faecium* SP15, a Potential Probiotic Strain Isolated from Spring Water. BMC Res. Notes.

[2] Klare I., Konstabel C., Badstübner D., Werner G., Witte W. (2003). Occurrence and Spread of Antibiotic Resistances in *Enterococcus faecium*. Int. J. Food Microbiol..

[3] Torres C., Alonso C.A., Ruiz-Ripa L., León-Sampedro R., Del Campo R., Coque T.M. (2018). Antimicrobial Resistance in *Enterococcus* spp. of Animal Origin. Microbiol. Spectr..

[4] Xuan H., Yao X., Pan R., Gao Y., Wei J., Shao D., Liu K., Li Z., Qiu Y., Ma Z. (2021). Antimicrobial Resistance in *Enterococcus faecium* and *Enterococcus faecalis* Isolates of Swine Origin from Eighteen Provinces in China. J. Vet. Med. Sci..

[5] Zaheer R., Cook S.R., Barbieri R., Goji N., Cameron A., Petkau A., Polo R.O., Tymensen L., Stamm C., Song J. (2020). Surveillance of *Enterococcus* spp. Reveals Distinct Species and Antimicrobial Resistance Diversity across a One-Health Continuum. Sci. Rep..

[6] Im E.J., Lee H.H.-Y., Kim M., Kim M.-K. (2023). Evaluation of Enterococcal Probiotic Usage and Review of Potential Health Benefits, Safety, and Risk of Antibiotic-Resistant Strain Emergence. Antibiotics.

[7] Krawczyk B., Wityk P., Gałęcka M., Michalik M. (2021). The Many Faces of *Enterococcus* spp.—Commensal, Probiotic and Opportunistic Pathogen. Microorganisms.

[8] Ferchichi M., Sebei K., Boukerb A.M., Karray-Bouraoui N., Chevalier S., Feuilloley M.G.J., Connil N., Zommiti M. (2021). *Enterococcus* spp.: Is It A Bad Choice A Good Use—A Conundrum Solve?. Microorganisms.

[9] Seputiene V., Bogdaite A., Ruzauskas M., Suziedeliene E. (2012). Antibiotic Resistance Genes and Virulence Factors in *Enterococcus faecium* and *Enterococcus faecalis* from Diseased Farm Animals: Pigs, Cattle and Poultry. Pol. J. Vet. Sci..

[10] Huang C., Moradi S., Sholeh M., Tabaei F.M., Lai T., Tan B., Meng J., Azizian K. (2025). Global Trends in Antimicrobial Resistance of *Enterococcus faecium*: A Systematic Review and Meta-Analysis of Clinical Isolates. Front. Pharmacol..

[11] Markwart R., Willrich N., Haller S., Noll I., Koppe U., Werner G., Eckmanns T., Reuss A. (2019). The Rise in Vancomycin-Resistant *Enterococcus faecium* in Germany: Data from the German Antimicrobial Resistance Surveillance (ARS). Antimicrob. Resist. Infect. Control.

[12] Arredondo-Alonso S., Top J., McNally A., Puranen S., Pesonen M., Pensar J., Marttinen P., Braat J.C., Rogers M.R.C., Van Schaik W. (2020). Plasmids Shaped the Recent Emergence of the Major Nosocomial Pathogen *Enterococcus faecium*. mBio.

[13] García-Solache M., Rice L.B. (2019). The *Enterococcus*: A Model of Adaptability to Its Environment. Clin. Microbiol. Rev..

[14] Guzman Prieto A.M., Van Schaik W., Rogers M.R.C., Coque T.M., Baquero F., Corander J., Willems R.J.L. (2016). Global Emergence and Dissemination of Enterococci as Nosocomial Pathogens: Attack of the Clones?. Front. Microbiol..

[15] Wei Y., Palacios Araya D., Palmer K.L. (2024). *Enterococcus faecium*: Evolution, Adaptation, Pathogenesis and Emerging Therapeutics. Nat. Rev. Microbiol..

[16] Wagner T., Joshi B., Janice J., Askarian F., Škalko-Basnet N., Hagestad O.C., Mekhlif A., Wai S.N., Hegstad K., Johannessen M. (2018). *Enterococcus faecium* Produces Membrane Vesicles Containing Virulence Factors and Antimicrobial Resistance Related Proteins. J. Proteom..

[17] Arias C.A., Murray B.E. (2012). The Rise of the *Enterococcus*: Beyond Vancomycin Resistance. Nat. Rev. Microbiol..

[18] Cattoir V., Giard J.-C. (2014). Antibiotic Resistance in *Enterococcus faecium* Clinical Isolates. Expert Rev. Anti Infect. Ther..

[19] Lee T., Pang S., Abraham S., Coombs G.W. (2019). Antimicrobial-Resistant CC17 *Enterococcus faecium*: The Past, the Present and the Future. J. Glob. Antimicrob. Resist..

[20] Yim J., Smith J.R., Rybak M.J. (2017). Role of Combination Antimicrobial Therapy for Vancomycin-Resistant *Enterococcus faecium* Infections: Review of the Current Evidence. Pharmacother. J. Hum. Pharmacol. Drug Ther..

[21] Gao W., Howden B.P., Stinear T.P. (2018). Evolution of Virulence in *Enterococcus faecium*, a Hospital-Adapted Opportunistic Pathogen. Curr. Opin. Microbiol..

[22] Zhou X., Willems R.J.L., Friedrich A.W., Rossen J.W.A., Bathoorn E. (2020). *Enterococcus faecium*: From Microbiological Insights to Practical Recommendations for Infection Control and Diagnostics. Antimicrob. Resist. Infect. Control.

[23] Gouliouris T., Coll F., Ludden C., Blane B., Raven K.E., Naydenova P., Crawley C., Török M.E., Enoch D.A., Brown N.M. (2020). Quantifying Acquisition and Transmission of *Enterococcus faecium* Using Genomic Surveillance. Nat. Microbiol..

[24] Cebeci T. (2024). Species Prevalence, Virulence Genes, and Antibiotic Resistance of Enterococci from Food-Producing Animals at a Slaughterhouse in Turkey. Sci. Rep..

[25] Messele Y., Hasoon M., Trott D., Veltman T., McMeniman J., Kidd S., Low W., Petrovski K. (2022). Longitudinal Analysis of Antimicrobial Resistance among *Enterococcus* Species Isolated from Australian Beef Cattle Faeces at Feedlot Entry and Exit. Animals.

[26] Ocejo M., Mugica M., Oporto B., Lavín J.L., Hurtado A. (2024). Whole-Genome Long-Read Sequencing to Unveil *Enterococcus* Antimicrobial Resistance in Dairy Cattle Farms Exposed a Widespread Occurrence of *Enterococcus lactis*. Microbiol. Spectr..

[27] Beukers A.G., Zaheer R., Goji N., Amoako K.K., Chaves A.V., Ward M.P., McAllister T.A. (2017). Comparative Genomics of *Enterococcus* spp. Isolated from Bovine Feces. BMC Microbiol..

[28] Brotto A.L.C., Silva S.V., Silva R.L.S., Antoniazzi F.B., Stievano J.M., Nakaghi A.C.H., Barberato-Filho S., Silva M.T., Bergamaschi C.C. (2022). Prevalence of antimicrobial-resistant *Enterococcus faecium* in commercial cattle: A systematic review and meta-analysis. Ars Vet..

[29] Makarov D.A., Ivanova O.E., Pomazkova A.V., Egoreva M.A., Prasolova O.V., Lenev S.V., Gergel M.A., Bukova N.K., Karabanov S.Y. (2022). Antimicrobial Resistance of Commensal *Enterococcus faecalis* and *Enterococcus faecium* from Food-Producing Animals in Russia. Vet. World.

[30] Messele Y.E., Trott D.J., Hasoon M.F., Veltman T., McMeniman J.P., Kidd S.P., Petrovski K.R., Low W.Y. (2023). Phylogeny, Virulence, and Antimicrobial Resistance Gene Profiles of *Enterococcus faecium* Isolated from Australian Feedlot Cattle and Their Significance to Public and Environmental Health. Antibiotics.

[31] Zaidi S.-Z., Zaheer R., Poulin-Laprade D., Scott A., Rehman M.A., Diarra M., Topp E., Domselaar G.V., Zovoilis A., McAllister T.A. (2023). Comparative Genomic Analysis of Enterococci across Sectors of the One Health Continuum. Microorganisms.

[32] Cinthi M., Coccitto S.N., Simoni S., Vignaroli C., Brenciani A., Giovanetti E. (2023). An *Enterococcus faecium* Isolated from Bovine Feces in Italy Shares *optrA*- and *poxtA*-Carrying Plasmids with Enterococci from Switzerland. Microb. Drug Resist..

[33] Lopes J., De Lencastre H., Conceição T. (2024). Genomic Analysis of *Enterococcus faecium* from Non-Clinical Settings: Antimicrobial Resistance, Virulence, and Clonal Population in Livestock and the Urban Environment. Front. Microbiol..

[34] Nurrahmat A.M.I., Susetya H., Putri K. (2025). Antibiogram Profile of *Enterococcus faecalis* and *Enterococcus faecium* in Chicken Meat from Supermarkets in Sleman District, Indonesia. Vet. World.

[35] Ryu H., Henson M., Elk M., Toledo-Hernandez C., Griffith J., Blackwood D., Noble R., Gourmelon M., Glassmeyer S., Santo Domingo J.W. (2013). Development of Quantitative PCR Assays Targeting the 16S rRNA Genes of *Enterococcus* spp. and Their Application to the Identification of *Enterococcus* Species in Environmental Samples. Appl. Environ. Microbiol..

[36] Clinical & Laboratory Standards Institute (2018). M100-Performance Standards for Antimicrobial Susceptibility Testing.

[37] Qiaoyun X., Mengzhi W., Yongjiu H., Yuhong L. (2018). Effects of *Enterococcus faecium* Preparation on Serum Biochemical, Antioxidant, and Immune Indices in Mid-Lactation Dairy Cows. Feed Ind..

[38] Fengqin W., Haitao X., Baocheng H., Xiaoyong X., Shijun B., Yonghao H. (2020). Isolation, Identification, and Biological Characterization of *Enterococcus faecium* from Sheep. Chin. J. Vet. Med. Herb. Med..

[39] Tiantian B., Xuefeng G. (2021). Characteristics of *Enterococcus faecium* and Research Progress on Its Application in Livestock Production. China Anim. Husb. J..

[40] Pengjuan G. (2016). Isolation and Identification of *Enterococcus faecium* Phage IME-EFm5 and Study on the Key Functional Sites of Its Endolysin. Ph.D. Thesis.

[41] Radford-Smith D.E., Anthony D.C. (2025). Vancomycin-Resistant, *E. faecium*: Addressing Global and Clinical Challenges. Antibiotics.

[42] Economou V., Gousia P. (2015). Agriculture and Food Animals as a Source of Antimicrobial-Resistant Bacteria. Infect. Drug Resist..

[43] Ghosh S., LaPara T.M. (2007). The Effects of Subtherapeutic Antibiotic Use in Farm Animals on the Proliferation and Persistence of Antibiotic Resistance among Soil Bacteria. ISME J..

[44] Hegstad K., Mikalsen T., Coque T.M., Werner G., Sundsfjord A. (2010). Mobile Genetic Elements and Their Contribution to the Emergence of Antimicrobial Resistant *Enterococcus faecalis* and *Enterococcus faecium*. Clin. Microbiol. Infect..

[45] Ahmad N., Joji R.M., Shahid M. (2023). Evolution and Implementation of One Health to Control the Dissemination of Antibiotic-Resistant Bacteria and Resistance Genes: A Review. Front. Cell. Infect. Microbiol..

[46] Hibbard R., Mendelson M., Page S.W., Ferreira J.P., Pulcini C., Paul M.C., Faverjon C. (2024). Antimicrobial Stewardship: A Definition with a One Health Perspective. Npj Antimicrob. Resist..

[47] McCubbin K.D., Barkema H.W., Babujee A., Forseille J., Naum K., Buote P., Dalton D., Checkley S.L., Lehman K., Morris T. (2022). One Health and Antimicrobial Stewardship: Where to Go from Here?. Can. Vet. J..

[48] Musoke D., Kitutu F.E., Mugisha L., Amir S., Brandish C., Ikhile D., Kajumbula H., Kizito I.M., Lubega G.B., Niyongabo F. (2020). A One Health Approach to Strengthening Antimicrobial Stewardship in Wakiso District, Uganda. Antibiotics.

[49] Mannu L., Paba A., Daga E., Comunian R., Zanetti S., Duprè I., Sechi L.A. (2003). Comparison of the Incidence of Virulence Determinants and Antibiotic Resistance between *Enterococcus faecium* Strains of Dairy, Animal and Clinical Origin. Int. J. Food Microbiol..

[50] Ribeiro T., Oliveira M., Fraqueza M.J., Lauková A., Elias M., Tenreiro R., Barreto A.S., Semedo-Lemsaddek T. (2013). Antibiotic resistance and virulence factors among *Enterococci* isolated from chouriço, a traditional Portuguese dry fermented sausage. J. Food Prot..

[51] Bin-Asif H., Ali S.A. (2019). The Genus *Enterococcus* and Its Associated Virulent Factors. Microorganisms.

[52] Jiahui W., Dan J., Hehai L., Aihong L., Guiquan G., Jianxun L., Hong Y., Youquan L. (2020). Detection of Virulence Genes and Antimicrobial Resistance in *Enterococcus faecium*. Chin. Vet. Sci..

